# Louisa Co. Med. Sc’y.

**Published:** 1857-06

**Authors:** T. G. Taylor

**Affiliations:** Sec’y, Pro tem.; Wapello, Iowa


### MEDICAL SOCIETIES.

Wapello, Iowa, April 24, 1857.

*Editors Iowa Medical Journal*:—At the annual meeting of the Louisa County Medical Society, held at this place on Saturday last, the election of officers for the ensuing year resulted as follows :

Dr. John Bell, President.

Wm. Cotton, Secretary.

Drs. J. M. Robertson, J. B. Latta, and H. T. Cleaver, Censors.

The day was chiefly spent in the report and discussion of cases of which several were of much interest.

Drs. Muldoon and Harley were admitted to membership in the Society.

Dr. Jno. Bell was elected delegate to the American Medical Association, and Dr. T. G. Taylor, delegate to State Medical Society.

Adjourned to meet on the second Tuesday in June next.

T. G. Taylor, Sec'y, *Pro tem.*

